# Food Choice Decisions of Collegiate Division I Athletes: A Qualitative Exploratory Study

**DOI:** 10.3390/nu13072322

**Published:** 2021-07-06

**Authors:** Kaitlyn M. Eck, Carol Byrd-Bredbenner

**Affiliations:** 1 Department of Nutrition and Dietetics, Marywood University, Scranton, PA 18509, USA; 2 Department of Nutritional Sciences, Rutgers University, New Brunswick, NJ 08901, USA; Bredbenner@sebs.rutgers.edu

**Keywords:** athletes, eating behaviors, sports nutrition

## Abstract

Limited research has examined athletes’ food and health beliefs and decisions and the congruence of these decisions with recommendations from nutrition professionals. This study aimed to improve understanding of athletes’ food-related beliefs and practices to enable nutrition professionals to more effectively enhance performance while protecting athletes’ health. Division I college athletes (n = 14, 64% female) from a variety of sports were recruited to participate in 20-min semi-structured phone interviews about food and nutrition-related behaviors and cognitions. Data were content analyzed to identify themes and trends. Prominent factors influencing athletes’ food choices were potential benefits to health and performance, availability of foods, and recommendations from sports dietitians. Foods commonly consumed by athletes, including fruits, vegetables, and lean protein, were generally healthy and aligned with sports nutrition recommendations. Athletes avoided energy-dense nutrient-poor foods, such as fast food and fried foods, with the goal of improving performance. Some athletes took supplements (i.e., multivitamin, iron, protein) on the premise that they would improve health and enhance performance or recovery. While athletes’ nutrition behaviors are generally congruent with current recommendations, findings highlighted misconceptions held by athletes related to the benefits of some supplements and the belief that packaged/processed foods were inherently less healthy than other options. Nutrition misconceptions held by athletes and incongruities between athletes’ nutrition knowledge and behaviors suggest that dietitians should aim to dispel misconceptions held by athletes and provide additional guidance and information to support athletes’ current healthful behaviors to ensure these behaviors extend beyond their college athletic career.

## 1. Introduction

Numerous factors influence food choices including cost, convenience, and taste. Food choices of athletes’ also may be influenced by actual and perceived effects of food on athletic performance, food preparation and eating time restraints, influence from coaches and teammates, and intra-sport culture (e.g., training and competition schedules, lifestyle choices affected by participation in sports) [1]. Younger athletes’ food choices may be affected by their observations of older, more experienced athletes who have the performance level and associated body shape the younger athlete is aiming to achieve [1]. Unfortunately, these experienced ‘role model’ athletes may not follow sports nutrition recommendations of the American College of Sports Medicine and the Academy of Nutrition and Dietetics [2].

Dietary recommendations associated with optimum performance vary by type of sport, with endurance athletes (e.g., distance runners, swimmers) requiring a greater proportion of calories from carbohydrates and strength athletes (e.g., powerlifters, track and field throwers [shot put, discus]) requiring more protein [3]. It is not uncommon for athletes to not meet dietary recommendations [4,5,6]. In some cases, athletes fail to meet the recommendations because the energy demands of practice and competition are so great that consuming enough food is difficult. In other cases, particularly in sports where leanness is desirable, athletes may intentionally undereat to reduce weight [7]. For instance, one study of female leanness sports athletes found that only 9% met calorie needs and 75% failed to reach recommended carbohydrate needs [8]. Similar results have been reported in male athletes [7]. Insufficient calorie intake leads to weight loss which may be beneficial to performance, however, intense training paired with restricted intake over a prolonged period of time ultimately negatively affects performance due to chronic incomplete glycogen repletion, incomplete recovery of muscle fibers between bouts of exercise, and micronutrient deficiencies [9].

On the other end of the spectrum are athletes who attempt to gain weight. Weight gain goals are common among athletes participating in sports emphasizing strength and power [10]. Although there is an abundance of weight loss information available, few publicly available sources report best practices for weight gain. The lack of information on healthy weight gain practices often results in athletes increasing calorie intake with high-fat foods rather than high-quality carbohydrates and protein as would be recommended by sports dietitians [11]. The lack of evidence-based information also may result in athletes relying on over-the-counter dietary supplements, some of which may contain substances that are prohibited by sports authorities and could lead to doping violations and/or harm to the athlete’s health [10]. 

Despite sports being a multi-billion-dollar enterprise and the clear relationship between diet and performance, limited research has focused on athletes’ food and health beliefs and choices, and the congruence of these choices with recommendations from nutrition professionals. A greater understanding of athletes’ food-related beliefs and practices is needed so that nutrition professionals can more effectively address beliefs and practices and promote changes to enhance performance while also protecting health. Thus, this study aimed to expand the understanding of factors influencing athletes’ food choice decisions by exploring athletes’ eating behaviors and the motivations behind these behaviors. It also aimed to explore athletes’ perceptions of healthy foods and healthy eating as well as their use of dietary supplements and the motivations behind supplement use.

## 2. Materials and Methods

The Authors’ University Institutional Review Board approved the study. All participants gave informed consent.

### 2.1. Sample 

Eligible participants were Division I college athletes at a large university; these athletes participate at the highest, most regulated level of intercollegiate athletics in the United States sanctioned by the National Collegiate Athletic Association [12]. Most Division 1 athletes are actively recruited for a collegiate sports team based on their athletic excellence in secondary education sports programs. They are among the most elite athletes and typically train up to 20 h per week [12]. Athletes were recruited via email announcements sent to coaches and athletic trainers and directly to athletes as well as in person at athlete fueling stations on campus and through referrals by athletes. Recruitment notices invited athletes to help researchers learn more about athletes’ eating behaviors. Students who participated in the study were entered into a drawing to receive 1 of 5 $25 gift cards as compensation for their time.

### 2.2. Instrument

A semi-structured interview guide was developed based on the results of interviews with dietitians working in the area of sports nutrition. The interviews gathered information on perceived behaviors of athletes and identified knowledge gaps related to athlete’s food/nutrition-related beliefs and behaviors. The interview guide was reviewed by a panel of 5 experts in the areas of sports nutrition, eating behaviors, and qualitative data collection.

A trained moderator conducted brief (~20 min) semi-structured phone interviews with each athlete while a trained note-taker recorded responses. Interviews were completed between September 2018 and August 2019. Interviews addressed 5 main topic areas: commonly consumed foods, avoided foods, food ‘rules’, their definition of healthy eating, and supplement use. Interview questions were developed de novo after a thorough literature review to identify gaps in the literature related to nutrition professionals’ understanding of athletes’ eating behaviors as well as results from a series of interviews with sports dietitians [13]. The semi-structured interview guide was followed closely to ensure consistent and complete data collection during each interview. Table 1 lists the interview questions.

### 2.3. Data Analysis

Within 24 h of completing each interview, notes were reviewed by the note-taker and edited for accuracy, clarity, and completeness. The notes were then reviewed by the moderator within 48 h of the interview to ensure accuracy and completeness. Any discrepancies in the notes between the note-taker and moderator were discussed, and a consensus was reached to finalize the notes. Then, a trained researcher qualitatively content analyzed the data. The constant analysis concurrent with subsequent interviews allowed the researcher to determine the point of data saturation (i.e., when no new information emerged from the interviews), which signaled data collection could end [14]. Once data saturation was reached, the data were independently content analyzed by two trained researchers using standard procedures [15,16] to identify themes and patterns within the data. Researchers compared their analyses and discussed differences to reach a consensus. The presentation of qualitative data is based on the verbal expressions of participants and “quotes are the primary form of evidence to support an author’s interpretation of the raw data” [17], thus this study employed the quote replete data reporting method.

## 3. Results

A total of 14 interviews were completed until data saturation was reached. All participants were Division I athletes and 64% female between the ages of 18 and 25 years. Athletes participated in a variety of sports including swimming (n = 3), track and field (n = 4), rowing, (n = 2), gymnastics (n = 2), tennis (n = 1), softball (n = 1), and volleyball (n = 1). The themes identified were based on the questions asked during the interview and centered around commonly consumed foods, avoided foods, their definition of healthy eating, and supplement use.

### 3.1. Commonly Consumed Foods

Findings from the athlete interviews are summarized in Table 1. In response to the interview question. “What foods or drinks do you consume every day or almost every day?”, nearly all athletes named water as a “staple” in their diet. Other foods frequently reported by athletes as being commonly consumed were chicken, fruits, vegetables, milk, Greek yogurt, and eggs. Athletes reported that fruits (e.g., apples and bananas) and snacks supplied by fueling stations at the gym (i.e., granola/protein bars, trail mix, fruit snacks) were common snacks.

When asked, “Why do you choose to consume these foods and drinks frequently?”, participants responded they drank water “to stay hydrated because we practice twice a day and I lose fluids”. Chicken and other sources of protein were consumed “for energy and muscle recovery”. The motivation for eating fruits and vegetables was “to make sure I have all the vitamins I need so my body is fully functioning”. Athletes chose to consume milk “because I want the calcium and protein source” and yogurt because “it’s great for probiotics and protein”. In general, athletes ate less processed foods because “they’re what makes me feel best as opposed to other more processed options”. Other factors influencing common food choices were “I like foods that are cheap and available” as well as foods that “fill me up”.

When asked, “What foods or drinks do you consume frequently because you feel they enhance your performance?”, most participants focused on the importance of hydration and noted that they are sure to drink plenty of water or Gatorade^®^ because “the more hydrated you are, the easier it is for your muscles to recover after your workout and you also just want to be hydrated throughout the day”. Athletes also reported that staying hydrated is key because “If I don’t have enough [fluids], it can affect my energy levels and staying hydrated helps athletes last longer through practice”. Protein was a key nutrient named by the athletes, many of whom reported choosing high protein foods because “we were told by our nutritionist that after practice we should consume protein”. Athletes believed that they “need protein to put on good muscle size”, that “protein shakes allow me to give the same intensity [to my workout] day after day”. Snacks like beef jerky, protein shakes, and chocolate milk were provided to the athletes after workouts which they perceived as supporting the importance of protein for recovery. Although the athletes identified protein-rich foods as performance-enhancing, they did not always know the specific benefits provided but relied on the advice of their sports nutritionist. Commenting about the benefits of chocolate milk one athlete said, “I forget why chocolate milk [is beneficial], but I’ve had nutritionists and trainers say it’s good for you”.

Eating during practice or competition was another theme that emerged. Athletes reported choosing fruit snacks or sports chews “if I get tired during practice” and want to “get some sugar in me” “to give me a little boost”. While some of the athletes reported they “don’t know what [these sports food products] have in them”, others stated that they had learned from the dietitian that these foods contain the “types of carbs that digest really quickly”.

Athletes felt specific nutrients that could be beneficial for performance. One athlete drank “milk for calcium because I’ve had injuries and I need strong bones”. Another reported being anemic and thus was “trying to eat spinach, take iron supplements, and eat high iron foods”. A distance runner shared that she “read somewhere that there are certain chemicals in beets that are good for distance running” and thus ate beets frequently.

Athletes also were asked, “What foods or drinks do you consume frequently because you feel they have a beneficial effect on your weight?”. Many reported that they were not concerned about their weight, but that fruits and vegetables are a good option for feeling full without overeating. A few athletes were trying to gain weight and reported that protein shakes and whole milk were some of the foods they ate often.

### 3.2. Avoided Foods

When asked, “What foods do you avoid?” and “Why do you avoid them?”, athletes readily named fast food, fried foods, and other foods that are “too processed, oily, or greasy”. Specific foods that met these criteria included french fries and pizza. Athletes avoided these because “I can feel it at practice the next day. I feel sluggish like it is sitting in my stomach”. Soda and other sugary drinks also were commonly named as being avoided. Some also reported avoiding sweets like candy and desserts.

When asked “What foods do you avoid while you are in season, but will eat out of season? Why are those foods reserved for the off-season?”, most athletes reported that there “isn’t too much of a difference” in their diet between the in and off-season. Some athletes were a bit more liberal during the off-season and had the occasional fast food meal or ate dessert more regularly. Alcohol was mentioned by two athletes as something that they avoid during the season, but do consume during the summer months when they are not practicing with the team. Some athletes reported that they “eat less healthy while in-season because, when we are traveling, sometimes we have to eat fast foods. Whereas, when I’m in the off-season, I’m always making my own food, my normal healthy stuff.”

In response to “What foods or drinks do you avoid because you feel they have a negative impact on your performance?”, athletes commonly named sugary drinks and fried foods. Athletes reported avoiding sugary drinks like soda and coffee drinks because they displaced water intake, which could negatively affect performance. They also avoided “heavy” or “fatty foods” like mac and cheese or pizza, particularly late at night because they can result in “feeling slow and sluggish” the next day.

When asked, “What foods or drinks do you avoid because you feel they would have a negative effect on your weight?”, sugary drinks and fatty food were again named. One athlete reported that it was important to limit sugary drink consumption because “over time, if the body doesn’t use up the sugars it consumes in a day, it stores it as fat.” Another athlete indicated it was important to select nutrient-dense foods over sugary treats noting, “calorie-wise, if I’m trying to get something out of it, I’d rather have some type of protein that builds muscle rather than something sugary like a Pop-Tart^®^”.

### 3.3. Definition of Healthy Eating

When asked, “How would you define healthy eating?”, the athletes repeatedly stated “balanced meals”, which they defined as “getting the food groups in each meal” or “having a balance of carbohydrates, protein, and vegetables”. Some athletes specified consuming 3 meals and at least a couple of snacks and “not skipping meals” was important. Variety (“a rainbow”) and portion size (not just what you eat, but how much) were also common responses. Some athletes specifically mentioned that it was important for athletes to focus on “eating enough” and noted that “cutting calories” or avoiding specific foods was not ideal for athletes.

In response to the question, “When you are deciding if a food is healthy or not, what types of things you think about?”, some athletes noted that they consider how the food will make them feel. Others reported talking to friends or their nutritionists about what constitutes healthy food. Most of the athletes felt unprocessed and home-cooked foods were healthier than processed foods and fast food because, “if I make it, it’ll be healthier because I know what’s going into it. I know what it is.” Many athletes said that “a lot of foods I consider healthy, I just know are healthy because they are what they are”, naming fruits, vegetables, lean meat, and whole grains as foods they considered to be healthy. A few athletes reported using the Nutrition Facts panel on processed foods to determine if was healthy. When reviewing the Nutrition Facts panel, these athletes reported noting the “fat content, specifically saturated fat” and “looking at the sugar content and the first few ingredients”.

Athletes were asked, “Do you think that healthy eating for an athlete is different than healthy eating for a non-athlete? How so?”. In general, the athletes felt that healthy eating for athletes and non-athletes were similar in some ways, but differed in others. The main difference noted by athletes was that they are eating to fuel performance, which can dictate their intake. As one athlete said, “the base [diet] is the same, but depending on what kind of athlete you are, you might have to increase the amounts of each particular [macronutrient] you need. It’s relatively similar, but higher calorically and content-wise for athletes.” For example, “for athletes, you need more protein at different points in the day” and “there are days before matches or any competition where athletes do need to carb load. Whereas, if a non-athlete carb-loaded, it wouldn’t be such a good idea.” The athletes noted that they consumed the “same types of food [as non-athletes], but larger portions.” Stating that, “You have to eat a lot more than the average person which makes it difficult because you feel like you are overeating, but that’s what you have to do.”

### 3.4. Supplement Use

Participants were asked, “What supplements, such as dietary or nutritional supplements, ergogenic aids, herbal supplements, vitamins, and minerals, if any, do you use? What is your reason for using the supplement?” The most common supplements were multivitamins, vitamin C, calcium, iron, and protein shakes. Other supplements included fish oil, glucosamine, echinacea, salt, and magnesium. The athletes who reported using a multivitamin said they did so as a “good way to get the vitamins I need”, reporting that it was convenient (“it takes two seconds and it’s easy to take”). Multivitamins were seen as a way to fill in dietary gaps as one athlete stated, “I know I still have to work on my diet. [I] want to make sure I’m getting some of the nutrients I might be missing from a multivitamin.” Vitamin C was thought to improve immune function and athletes reported “Vitamin C keeps us well, sickness affects our training. I don’t want to get sick and miss training or have it mess up our performance.” Athletes who reported taking calcium supplements did so to strengthen bones and prevent stress fractures that can take them out of competition. Most athletes who reported taking iron reported that they had “been tested for low iron and if I’m feeling particularly tired, or sluggish, I’ll get my blood drawn before I take iron.” However, one athlete noted that “a bunch of kids on our team have an iron issue, more in the women obviously but some of the men, but I take iron pills because I don’t want to be one of the kids with an iron deficiency.” Post-workout protein shakes (or high protein milk drinks) were the most common supplement used by athletes who wanted to “start rebuilding muscle right after practice” in order to “feel like I’m getting the maximum out of the workout”. Although some athletes reported that they “can’t remember the science behind it, but I know it does help with recovery, someway somehow.” They felt these shakes were beneficial because “it’s what [the nutrition staff] provide us in our fridge [in the weight room].” All athletes reported consuming at least one supplement. However, an athlete who only consumed protein shakes and Emergen-C^®^ stated, “I don’t take too many supplements because I forget or I don’t necessarily know if I’m lacking in anything and need them.”

## 4. Discussion

This qualitative study of athletes’ food behaviors and influences found that while some behaviors and beliefs held by athletes are in line with current sports nutrition recommendations (i.e., the inclusion of fruits, vegetables, and lean protein, and avoidance of energy-dense nutrient-poor foods) and are supported by the most recent research, other behaviors and cognitions are not (i.e., supplement use, perception of processed foods). Previous studies have identified several factors that influence athletes’ food choices including nutrition knowledge, preference, cost, convenience, and influences from coaches and teammates [1]. Some of these factors, including cost, preference, and nutrition knowledge, were found to be influential in this study population. However, this qualitative study identified other prominent factors that influenced collegiate athletes’ food choices, including potential benefits to health (e.g., high vitamin and mineral content in fruit, probiotics in yogurt) and performance (e.g., lean protein for muscle recovery).

The athletes reported avoiding “unhealthy” foods (i.e., high fat and high sugar foods) because they made them feel lethargic and inhibited their ability to perform well at practice or in competition, which is congruent with other research [1]. Interestingly, few athletes stated they avoided these “unhealthy” foods because they were high in calories or had the potential to cause weight gain. No athletes reported avoiding entire food groups (e.g., dairy) or specific food components (i.e., gluten), which contrasts with practices commonly reported in the literature [18,19]. Previous studies have relied on surveys to collect data, thus there is the potential for the different study designs (qualitative vs. quantitative) to explain these differences in findings. The finding that athletes in this study reported very little variation in their diet between the on and off-season also contrasts with findings from studies with college football players [1] and female college athletes [20,21]. These differences may be because the athletes did not perceive that they altered the type of foods they consumed during the sports season cycle, however, changes in the amount of food and intuitive shifts in macronutrient distribution likely occurred to support the needs of training and competition.

A contradiction in the athletes’ beliefs and behaviors was observed. Many of the athletes believed that processed foods were less healthy than fresh foods prepared at home. However, processed foods like shakes, bars, and fruit chews were commonly eaten. It is possible that the athletes were more accepting of the processed foods served at athlete fueling stations because they had been approved by the sports dietitian. This suggests that athletes may benefit from education from dietitians on how to evaluate foods nutritionally. Further, dietitians can correct misconceptions related to “processed” foods and nutritional value. While the qualitative methodology used in this study expands on other quantitative studies [22,23], future research should further explore athlete’s perceptions of processed foods and the potential conflict between perceived healthfulness and convenience.

Athletes obtain nutrition information from a variety of sources including teammates, trainers, and nutritionists. In this study, nutritionists had a strong influence on dietary choices in that athletes often reported selecting foods or beverages because their nutritionist recommended them or they were available at the athlete fueling stations and perceived to be healthy because they were handpicked for the athletes by the nutrition team. This environmental control of athletes’ intake suggests that the athletes learn eating behaviors from the foods provided at the fueling stations. Thus, sports dietitians should aim to provide high-quality foods at these locations, while building on athletes’ perceptions of healthy eating to teach them how to purchase and select healthy foods on their own. This will benefit athlete’s long-term and may promote health and wellbeing in the post-collegiate years.

In some cases, the athletes’ knowledge of the benefit of a food or beverage product they ate at the recommendation of the nutritionist was limited, but they consumed it because their nutritionist recommended it. This suggests that sports dietitians are influencing athletes’ behaviors, but not their knowledge. Future interventions may benefit by aiming to improve both knowledge and behavior by teaching athletes not just what to consume, but why it is a healthy choice and when and how much of each food to consume. The knowledge athletes gain can be utilized to support health and well-being after their collegiate athletic career has ended.

Nutritional needs of athletes differ from the general population, with athletes generally having higher energy needs due to the demands of training and competition [1,24]. The athletes in this study were aware of this difference and reported energy needs to be the main difference between a healthy diet for an athlete and a non-athlete. Numerous studies have found that athletes fail to meet energy needs [4,5,6]. The findings of this study suggest that inadequate intake is not a result of a lack of knowledge about increased energy demands. Numerous other physiological barriers have been identified [25]. Future studies should further explore these barriers. Other studies have reported discrepancies between athletes’ perceived and actual intake [26,27], which may in part explain the inconsistency between knowledge and behavior. Sports dietitians should educate athletes to help them reduce the discrepancy between perceived and actual intake.

The strengths of this study lie in the methodology employed. Most studies use quantitative methods which have the shortcomings of failing to consider an individual’s view-points and the “why” behind behaviors and cognitions [28]. Qualitative data methods were utilized to explore athletes’ nutrition practices and beliefs because this methodology allows researchers to conduct in-depth probing of a topic to uncover nuances that cannot be captured in quantitative data collection, such as surveys [28]. To achieve the benefits of qualitative methods, careful attention was given to instrumentation, data collection, and data analysis. The semi-structured interview guides were designed to address literature gaps and to build on qualitative interviews with sports dietitians and were followed closely to ensure uniform data collection. Further consistency in data collection was achieved in that the same trained moderator conducted all interviews. Another strength was that interview data were continuously analyzed to ensure data collection continued until data saturation occurred. Study strengths must be considered in light of the limitations. Participants were limited to Division I athletes at a single university, thus it is not known whether the findings are generalizable to similar athletes in other geographic regions. These athletes also compete at a very high level and have access to sports dietitians and other sport and health professionals, and thus the findings likely cannot be extrapolated to less competitive athletes with less access to supports. The sample size could be considered a study limitation, particularly given the fact that the sample included athletes from a variety of sports. However, data collection continued until data saturation was achieved. The point of data saturation (the point at which no new trends or themes emerged) is the marker used in qualitative research to indicate when data collection should cease. The results of this exploratory study provide insights for future research, which may include further in-depth qualitative investigation of differences by sport-type and/or competition level as well as inform the development of quantitative research methods (e.g., surveys) that examine differences in athletes’ food choice decisions by age, years in sport, type of sport, and nutrition knowledge level.

Sports dietitians are concerned mainly with promoting the performance and recovery of athletes [2]. However, the findings of this study indicate there many opportunities for dietitians to augment and extend their influence by building athletes’ nutrition knowledge about the food choices recommended and offered and dispel misconceptions such as those related to processed foods and supplements. Athletes view sports nutritionists as a trusted source of information, thus by using a ‘teachable moment’ in their lives, sports nutritionists have the opportunity to benefit athletes now as well as extend their influence into athletes’ future post-competition years to help retired athletes chose a healthy diet to avoid the weight gain and associated comorbidities that some, especially football players, experience [29].

## 5. Conclusions

The qualitative design of this study has extended the understanding of athletes’ eating behaviors by permitting in-depth explorations of cognitive factors impacting behaviors and perceptions identified in quantitative research. As an example, the qualitative study identified influences on food choices made by athletes that, to the best of the authors’ knowledge, have not been examined in quantitative surveys, such as athletes’ perceptions that their intake did not differ by sports season cycle, which is in direct contrast to quantitative studies indicating intake does differ [1,20,21]. Future mixed methods studies should aim to identify the cause of this discordance between perceptions and reality as well as implications for dietary quality and health outcomes. The qualitative design of the study also highlighted incongruities that exist between athletes’ nutrition knowledge (i.e., energy needs, nutrient needs, healthy food choices for athletes) and behaviors, suggesting a need for interventions that bring these into congruence. The findings of this study have the potential to help sports dietitians and other health professionals address gaps and incongruities that have the potential to lead to healthier, more informed athletes now and in the future.

## Figures and Tables

**Table 1:** Semi-structured Interview Items and Summary of Eating Behaviors of Division I Athletes (n = 14).

Question: “What foods or drinks do you consume every day or almost every day?” and “What foods or drinks do you consume frequently because you feel they enhance your performance?”	
Responses	
**Commonly Consumed Food**	**Reason for Consuming**
Water	Hydration
Lean protein (i.e., chicken, eggs)	Protein for recovery
Fruits and vegetables	High in vitamins and minerals Low in calorie/filling
Milk	Protein and calcium
Greek yogurt	Protein and probiotics
Granola bars/sports bars	Convenient, quick fuel
**Question: “Why do you choose to consume these foods and drinks frequently?”**	
**Responses**	
Availability (provided at fueling station)	
Promoted by dietitian	
Preference	
Cost	
**Question: “What foods do you avoid?” and “Why do you avoid them?”**	
**Responses**	
**Avoided Foods**	**Reason for Avoiding**
Greasy/fatty food (i.e., fast food, fried food, pizza)	Improve performance
Soda	Improve energy
Added sugar/sweets (i.e., candy, dessert)	Control weight
Alcohol	
**Question: “What foods do you avoid while you are in season but will eat out of season? Why are those foods reserved for the off-season?”**	
**Responses**	
Few changes overall	
Some eat more liberally (i.e., more greasy foods or sweets, more alcohol)	
**Question: “What foods or drinks do you consume frequently because you feel they enhance your performance?”**	
**Responses**	
Consuming a “go-to” meal before a competition (i.e., pasta dinner)	
Fueling during a competition (i.e., gels, fruit chews)	
**Question: “How would you define healthy eating?”**	
**Responses**	
Balanced meals (i.e., all food groups)	
Not skipping meals	
Variety (i.e., do not eliminate any one food group/nutrient)	
Healthy portions (focus on eating enough)	
**Question: “Do you think that healthy eating for an athlete is different than healthy eating for a non-athlete? How so?”**	
**Responses**	
Generally similar	
Athletes eat to perform not just to stay healthy	
Athletes have higher calorie needs	
**Question: “What supplements, such as dietary or nutritional supplements, ergogenic aids, herbal supplements, vitamins, and minerals, if any, do you use? What is your reason for using the supplement?”**	
**Responses**	
**Supplement**	**Reason for Consuming**
Micronutrient supplements (i.e., multivitamins, iron, calcium, fish oil)	Fill dietary gaps
Vitamin C	Improve immune function
Iron	Enhance performance
Calcium	Prevent injury
Protein supplements (i.e., protein shakes, ultra-filtered milk)	Promote recovery

## Data Availability

Data summarized in this study is available from the corresponding author upon request.
